# Metformin and atorvastatin ameliorate MASH via the AMPK/NF-κB pathway

**DOI:** 10.3389/fendo.2026.1972846

**Published:** 2026-10-12

**Authors:** Bin Zhang, Na Li, Fang Liu, Guorui Wang, Quanwei Chen, Yiming Sun, Ni Yang, Hao Zhang, Jianjun Han

**Affiliations:** 1 Department of Interventional Therapy Center, Shandong Cancer Hospital and Institute, Shandong First Medical University and Shandong Academy of Medical Sciences, Jinan, Shandong, China; 2 Department of Radiology, Guang’anmen Hospital Jinan Branch, Jinan Municipal Hospital of Traditional Chinese Medicine, Jinan, Shandong, China; 3 Department of Radiology, Shandong Cancer Hospital and Institute, Shandong First Medical University and Shandong Academy of Medical Sciences, Jinan, Shandong, China

**Keywords:** atorvastatin, inflammation, lipid metabolism, MASH, MASLD, metformin

## Abstract

**Background:**

Despite being a globally prevalent chronic liver disease, MASLD currently lacks any approved pharmacotherapy. MET and ATO individually confer benefits against MASLD, but their combined therapeutic effects have seldom been investigated. The current work aimed to verify the therapeutic benefits of their combined intervention on MASH.

**Methods:**

A MASH-like mouse model was established via HFD feeding combined with CCl_4_ injection, alongside an FFA-induced HepG2 steatosis model. Serum biochemical markers and inflammatory cytokines were measured, and lipid deposition was assessed by Oil Red O staining. Western blotting was performed to evaluate the expression of lipid metabolism-related proteins and AMPK/NF-κB signaling pathway components. Additionally, Compound C, a pharmacological inhibitor of AMPK, was used to verify the underlying molecular mechanism.

**Results:**

Co-treatment with MET and ATO markedly alleviated FFA-induced lipid accumulation and TG/TC levels in HepG2 cells. Combination therapy in HFD/CCl_4_-induced MASH-like mice reduced body/liver weights, fasting glucose and HOMA-IR. Mechanistically, it downregulated lipogenic proteins SREBP-1 and HMGCR while upregulating fatty acid oxidation regulators CPT1A and PPARα. Additionally, it suppressed IL-6, TNF-α and IL-1β secretion via inhibiting NF-κB phosphorylation, thereby mitigating hepatic and cellular inflammation. Moreover, the combination enhanced AMPK phosphorylation, while the AMPK inhibitor Compound C largely abolished these protective effects.

**Conclusion:**

These observations indicate that the combined MET and ATO treatment exerts lipid-lowering and anti-inflammatory effects possibly via the AMPK/NF-κB signaling axis.

## 1 Introduction

With a global prevalence of nearly 25%, Metabolic dysfunction‑associated steatotic liver disease (MASLD) stands as one of the most common chronic hepatic disorders across the world (1). As obesity and type 2 diabetes keep growing more prevalent worldwide, the incidence of MASLD is rising steadily and is anticipated to rank as the most common indication for liver transplantation resulting from end-stage liver disease (2). This disorder progresses along a pathological spectrum that stretches from isolated hepatic steatosis, MASH and liver fibrosis to cirrhosis and eventually hepatocellular carcinoma (HCC); however, no approved disease-specific pharmacological therapies are currently available (3). The “multiple-hit” hypothesis provides the best framework for understanding the complex pathogenesis of MASLD, with hepatic lipid metabolism dysregulation and chronic inflammation considered core pathological contributors, which are also the two major aspects that this study focuses on (4).

The progression of MASLD is driven by a vicious cycle between disordered lipid metabolism and chronic inflammation. On the one hand, excessive accumulation of free fatty acids in hepatocytes activates inflammatory signaling pathways through lipotoxicity (5). On the other hand, by releasing IL-6, TNF-α, and IL-1β upon NF-κB activation, pro-inflammatory cytokines further impair fatty acid oxidation and accelerate *de novo* lipogenesis, creating a detrimental feedback loop that pushes the pathological progression from steatosis toward steatohepatitis (6–8). As a key regulatory molecule of energy metabolism, AMPK not only inhibits fatty acid synthesis and promotes fatty acid oxidation, but also attenuates inflammatory responses by suppressing the NF-κB pathway, serving as a “molecular switch” linking lipid metabolism and inflammation. Its activity is often impaired in the MASLD state, making it a highly promising therapeutic target (9).

Metformin (MET) and atorvastatin (ATO) are two widely prescribed agents. As a biguanide, MET exerts hypoglycemic, anti-inflammatory, and anti-adipogenic effects and is widely used as first-line therapy for type 2 diabetes and obesity, mainly through activating the AMPK cascade to suppress hepatic gluconeogenesis, promote fatty acid oxidation, and attenuate inflammation (10). ATO, a statin, delivers hypolipidemic and anti-inflammatory actions via inhibition of HMG-CoA reductase, which restrains endogenous cholesterol synthesis (11, 12). Accumulating evidence indicates that ATO may modulate AMPK signaling, contributing to its metabolic and anti-inflammatory benefits (13). Notably, both agents improve lipid metabolism and attenuate inflammation through the AMPK pathway, whose activation suppresses NF-κB-dependent inflammatory signaling (14, 15). Although separate studies have documented the favorable effects of MET and ATO against MASLD, the therapeutic efficacy of their combination and the underlying mechanisms remain to be systematically explored.

Both *in vivo* and *in vitro* experiments were performed in this work, using a murine HFD/CCl_4_-induced MASH-like model and an FFA-stimulated HepG2 cellular system, with the aim of systematically evaluating how combined MET and ATO administration affects lipid metabolism, inflammatory responses, and the AMPK/NF-κB pathway. Furthermore, the AMPK inhibitor Compound C was employed to verify the dependence on this pathway. The present investigation seeks both to elucidate the mechanistic basis of MET and ATO combination therapy for MASH and to foster its clinical adoption.

## 2 Methods

### 2.1 Cell culture and treatment

Obtained from the China Center for Type Culture Collection (Wuhan, China), HepG2 cells were grown in Gibco high-glucose DMEM supplemented with 10% FBS and 1% penicillin-streptomycin (Biosharp) at 37 °C under 5% CO_2_ with humidity. To prepare the FFA working solution, oleic acid and palmitic acid (Puxitang, China) were blended at a 2:1 molar ratio and conjugated with 0.1 mol/L NaOH solution containing 1% fatty acid-free BSA. After heating at 60 °C for 30 min, the solution was filter-sterilized (0.22 μm), and the final pH was adjusted to pH 7.4. The FFA stock solution was then diluted with complete medium to a final working concentration of 0.3 mmol/L for subsequent experiments on HepG2 cells. For FFA exposure, HepG2 cells were seeded at a density of 2 × 10^5^ cells/well in 6-well plates and cultured until approximately 80% confluence. The cells were then exposed to the FFA working solution at a final concentration of 0.3 mmol/L for 24 h. The exposure medium was complete medium with serum, and all experiments were performed independently in triplicate (16).

Cells were divided into five groups: Control, FFA, FFA+MET, FFA+ATO, and FFA+MET+ATO. To verify AMPK pathway involvement, an additional group was included, in which cells were pretreated with 10 μmol/L Compound C (Selleck, China) for 1 h before drug exposure (17). All treatments were performed for 24 h.

### 2.2 Cell viability assay

Following the manufacturer’s guidelines, the CCK-8 kit (Solarbio, China) was used to detect cellular viability. For cell attachment experiments, HepG2 cells were seeded into 96-well plates at 5×10³ per well and incubated at 37 °C under humidified 5% CO_2_ conditions until they adhered completely. Upon 24h treatment, 10 μL CCK‑8 was added to each well and incubated at 37 °C in the dark for 1 h. A microplate spectrophotometer was applied to detect absorbance values at 450 nm. Cell viability was calculated relative to the untreated cell control group (cells cultured in complete medium without drug intervention) using the following formula: cell viability (%) = [(A-sample – A-blank)/(A-control – A-blank)] × 100%, where A-sample is the absorbance of drug-treated cells, A-control is the absorbance of untreated cells, and A-blank is the absorbance of wells containing complete medium and CCK-8 reagent without cells.

### 2.3 Oil Red O staining

Upon termination of the treatments, the culture medium was aspirated, and the cells underwent one gentle rinse with PBS. Subsequently, cells were fixed with 4% paraformaldehyde for 30 min at RT, briefly rinsed with 60% isopropanol for 15 s, and washed with PBS. Subsequently, freshly filtered Oil Red O staining solution (5 mg/mL; Solarbio, China) was added to samples, which were incubated for 15 min at ambient temperature under light‑shielded conditions. Finally, the excess stain was eliminated by a 15-s rinse with 60% isopropanol, followed by a PBS washing step.

An upright light microscope (Nikon, Japan) fitted with Nikon’s image acquisition system was used to observe and photograph intracellular lipid droplets. Intracellular lipid accumulation was quantitatively analyzed using ImageJ software.

### 2.4 Animal experiments and experimental design

SPF-grade male C57BL/6J mice, aged 6 weeks and purchased from Beijing SPF Biotechnology Co., Ltd., were kept in an environment with 22 ± 2 °C temperature, 55 ± 5% relative humidity, and a 12-hour light/dark photoperiod. Food and water were supplied ad libitum throughout the seven‑day acclimation period. All mice had free access to standard sterile water throughout the experimental period. No additional reagents, supplements, or drugs were added to the drinking water. Water was provided ad libitum in standard drinking bottles and replaced twice weekly. All procedures were approved by the Laboratory Animal Ethics Committee of Shandong Cancer Hospital (Approval No. SDTHEC202411002).

A total of 30 mice were used. Following adaptive feeding, mice were randomized into a normal control group (n=6) and a high-fat diet (HFD) group (n=24) fed a diet containing 60% kcal from fat (PD6001, Changzhou SYSE Bio-Tec. Co., Ltd., China; 60% fat, 20% protein, and 20% carbohydrate by energy) for 12 week. The HFD-fed mice were then re-randomized into four subgroups (n=6 each): HFD, HFD+MET, HFD+ATO, and HFD+MET+ATO. During the subsequent 8-week intervention, metformin (200 mg/kg/day) and/or atorvastatin (10 mg/kg/day) were administered by oral gavage, while controls received saline (18, 19). During the first 4 weeks of this 8-week treatment period, all HFD-fed mice received intraperitoneal injections of 10% CCl_4_ in olive oil (0.5 mL/kg) once weekly to accelerate the progression from simple steatosis to MASH-like pathology, as previously reported (20). The normal control group received a normal diet and an equivalent volume of olive oil alone (without CCl_4_) via the same route and schedule.

Body weight and food intake were recorded weekly. At the end of the experiment, blood and liver samples were collected for subsequent biochemical and histopathological analyses.

### 2.5 Fasting blood glucose and insulin measurements

A commercial glucose assay kit (S03039, Leidu, China) was employed to determine fasting blood glucose (GLU) levels, and a mouse insulin ELISA kit (CEA448Mu, Youersheng Biotechnology, China) was used to measure fasting insulin (INS) concentrations. The HOMA-IR index was then applied to evaluate insulin resistance, with the formula: HOMA-IR = GLU (mmol/L) × INS (mIU/L)/22.5.

### 2.6 Biochemical analysis

An automatic biochemical analyzer was employed to quantify serum ALT, AST, TG, TC, HDL‑C, and LDL‑C. Triglyceride and total cholesterol levels in hepatic tissues and cultured cells were measured with Nanjing Jiancheng Bioengineering Institute kits per attached manuals. All assays followed unified standard protocols. Serum and HepG2 supernatant IL-6, IL-1β, and TNF-α concentrations were determined using ELISA kits (Jingmei, China).

### 2.7 Histopathological analysis

To perform histopathological observation, liver samples were subjected to fixation using 4% paraformaldehyde followed by paraffin embedding, and then cut into sections with a thickness of 5 μm. Subsequently, the tissue slices underwent xylene dewaxing, gradient ethanol rehydration, and conventional H&E staining according to standard laboratory procedures. To visualize neutral lipid deposition, OCT-embedded frozen hepatic slices with a thickness of 10 μm were dyed with Oil Red O, followed by hematoxylin counterstaining. Lipid accumulation was viewed under a light microscope, with representative images recorded. The NAS system was applied to grade histological severity, evaluating steatosis (0-3), lobular inflammation (0-2), and ballooning (0-2); the overall score was the sum of these components. In line with standard criteria, NAS ≥5 indicated MASH, whereas NAS <3 ruled out MASH. Histological evaluation was performed by two independent blinded pathologists on one full liver section per animal, and the average score generated by the two assessors was utilized for statistical analysis (n = 6 per group).

### 2.8 Western blot analysis

Hepatic tissue and HepG2 cell total proteins were extracted using inhibitor‑supplemented RIPA buffer and purified by centrifugation (13000 rpm, 4 °C, 15 min). Protein content was determined using BCA kits (Addison, China). Aliquots containing 30 μg protein were separated by SDS‑PAGE and electrotransferred to PVDF membranes. Membranes were blocked with rapid blocking buffer (Aoru, China) for 1 h at room temperature, then incubated overnight at 4 °C with primary antibodies against p-AMPK (1:1000; Cat. No. YP-Ab-14296; Rabbit), AMPK (1:1000; Cat. No. YP-mAb-14656; Mouse), SREBP1 (1:1000; Cat. No. YP-mAb-10754; Mouse), HMGCR (1:1000; Cat. No. YP-mAb-17137; Mouse), CPT1A (1:1000; Cat. No. YP-mAb-08231; Mouse), and PPARα (1:1000; Cat. No. YP-mAb-03324; Mouse) (UpingBio); p-NF-κB (1:2000; Cat. No. 80379-2-RR; Rabbit) and NF-κB (1:1000; Cat. No. 10745-1-Ap; Rabbit) (Proteintech); and GAPDH (1:10,000; Cat. No. bs-2188R; Rabbit) and Tubulin (1:5,000; Cat. No. bsm-33034R; Rabbit) (Bioss). The blots, after being washed three times with TBST (10 min each), were exposed to HRP-linked secondary antibodies (goat anti-rabbit, Cat. No. BE02187352; goat anti-mouse, Cat. No. BE02106441; 1:5000, Bioss) for 60 min at RT. Protein bands were visualized using an ECL kit (Biosharp, China) and quantified with ImageJ. For total proteins: Band intensity of each target protein was normalized to its loading control (Tubulin or GAPDH) to generate group-wise target/control ratios. Relative expression was calculated by dividing each ratio by the corresponding value from the control group. For phosphorylated proteins: Band intensities were normalized against matched total protein bands to get p-protein/total protein ratios. These ratios were referenced to the control group to determine relative phosphorylation levels. The cropped blots are presented in the figures; full-length original blots are available in the **Supplementary Figure 1**.

### 2.9 Statistical analysis

The trials were replicated autonomously at least three occasions. Measured outcomes across experiments are reported as mean ± SEM. GraphPad Prism 9.0 (San Diego, CA, USA) served for statistical data analysis. Unpaired two‑tailed Student’s t‑tests were adopted for pairwise comparisons between two cohorts. For analyses covering three or more experimental subgroups, one‑way ANOVA combined with subsequent post‑hoc analyses was adopted to assess intergroup differences. We considered any findings to be statistically significant if they reached a P-value below 0.05. To denote levels of significance, we used asterisks: *for *P* < 0.05, **for *P* < 0.01, ***for *P* < 0.001, ****for *P* < 0.0001, and ns for anything that didn’t reach statistical significance.

## 3 Results

### 3.1 Combined metformin and atorvastatin treatment alleviates free fatty acid-triggered lipid deposition in HepG2 cells

To explore how MET and ATO affect intracellular fat accumulation triggered by excess free fatty acids within HepG2 hepatocytes, the CCK8 test was initially applied to detect cell viability. As depicted in **Figures 1A, B**, treatment with 2 mM MET or 10 μM ATO did not notably reduce cell viability (>90%), and thus these concentrations were chosen for further studies. To eliminate the possible confounding effects of cytotoxicity on experimental findings, we assessed cell viability of all treatment groups (including FFA, FFA+MET, FFA+ATO, FFA+MET+ATO, and Compound C-containing groups) with the CCK-8 test. The data revealed that cell survival stayed above 80% across all groups (**Supplementary Figure 2**). Oil Red O dye staining revealed extensive intracellular lipid accumulation once the HepG2 cells underwent free fatty acid stimulation, whereas treatment with either MET or ATO alone reduced lipid droplet deposition. The combined treatment produced the greatest reduction in lipid accumulation (**Figures 1C, D**). In line with the above results, free fatty acid incubation significantly elevated intracellular triglyceride (TG) and total cholesterol (TC) concentrations in hepatocytes. Separate treatment with metformin or atorvastatin lowered these lipid parameters, and co-administration generated the optimal hypolipidemic outcome (**Figures 1E, F**). Subsequent western blotting detection demonstrated that compared with cells only treated with free fatty acids, combined medication significantly suppressed the expression of lipogenic-related proteins SREBP-1 and HMGCR. Meanwhile, this dual-drug regimen upregulated two critical modulators governing fatty acid oxidation, namely CPT1A and PPARα (**Figures 1G–K**). Collective data suggest that co-administration of MET and ATO can robustly alleviate metabolic disorders of lipids triggered by free fatty acids within HepG2 hepatocytes.

**Figure 1:**
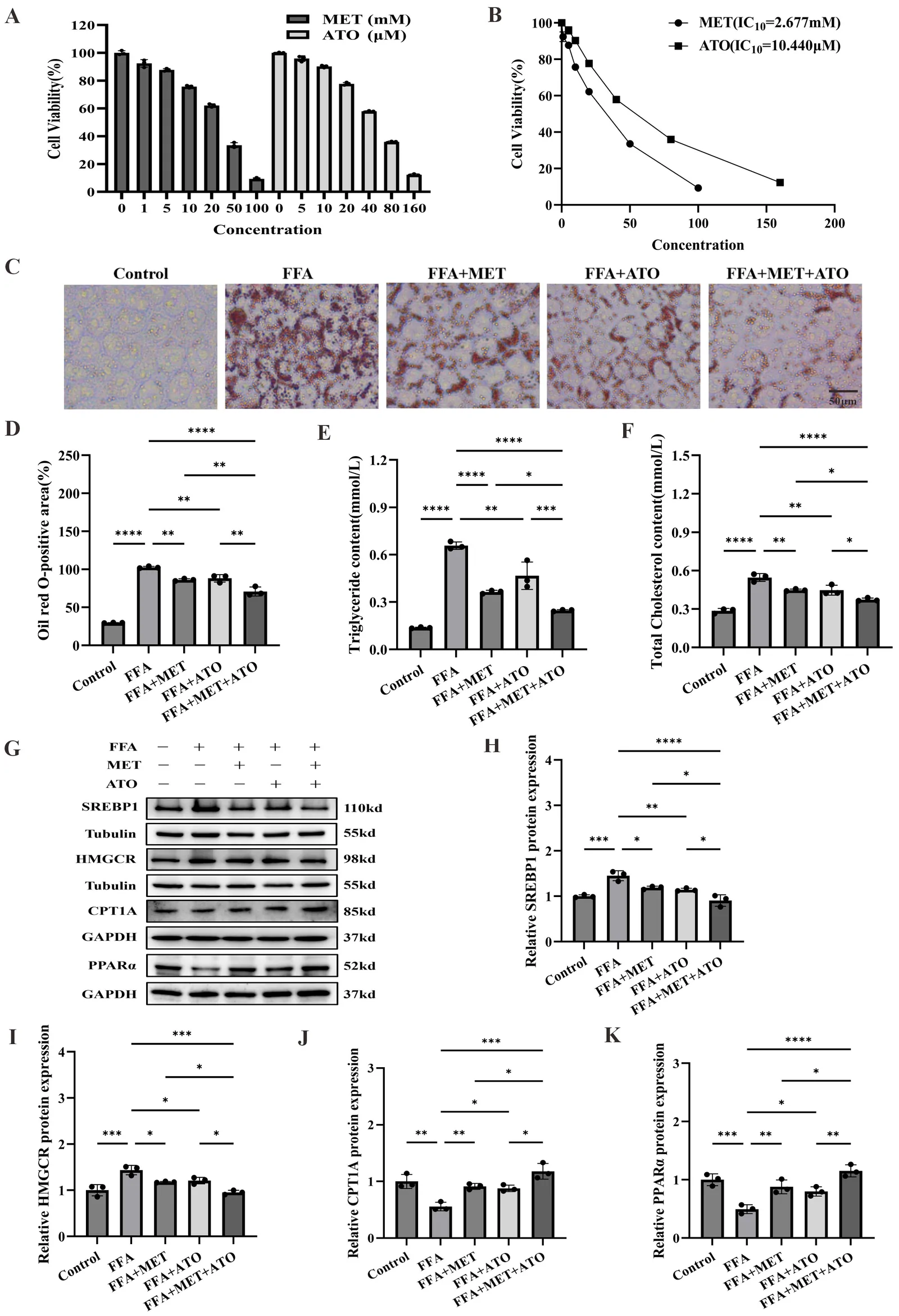
Displays that MET plus ATO co-administration attenuates FFA-triggered lipid buildup in HepG2 cells. **(A, B)** Assessment of cell viability. **(C)** Oil Red O staining of cells across groups (40×; scale bar = 50 μm). **(D–F)** Quantitative analysis of Oil Red O staining as well as the detection of TG and TC contents. **(G–K)** Western blotting for SREBP1, HMGCR, CPT1A, and PPARα protein levels. All values are presented as mean ± SEM (n = 3). *P < 0.05, **P < 0.01, ***P < 0.001, ****P < 0.0001.

### 3.2 Combined metformin and atorvastatin treatment attenuates FFA-induced inflammatory responses in HepG2 cells

ELISA was employed to determine the levels of IL-6, TNF-α, and IL-1β in cell culture supernatants, in order to evaluate the anti-inflammatory activity of MET and ATO. Compared with the control, FFA substantially increased these cytokines (**Figures 2A–C**). MET or ATO alone each lowered their levels, whereas co-administration achieved the maximum inhibition.

**Figure 2:**
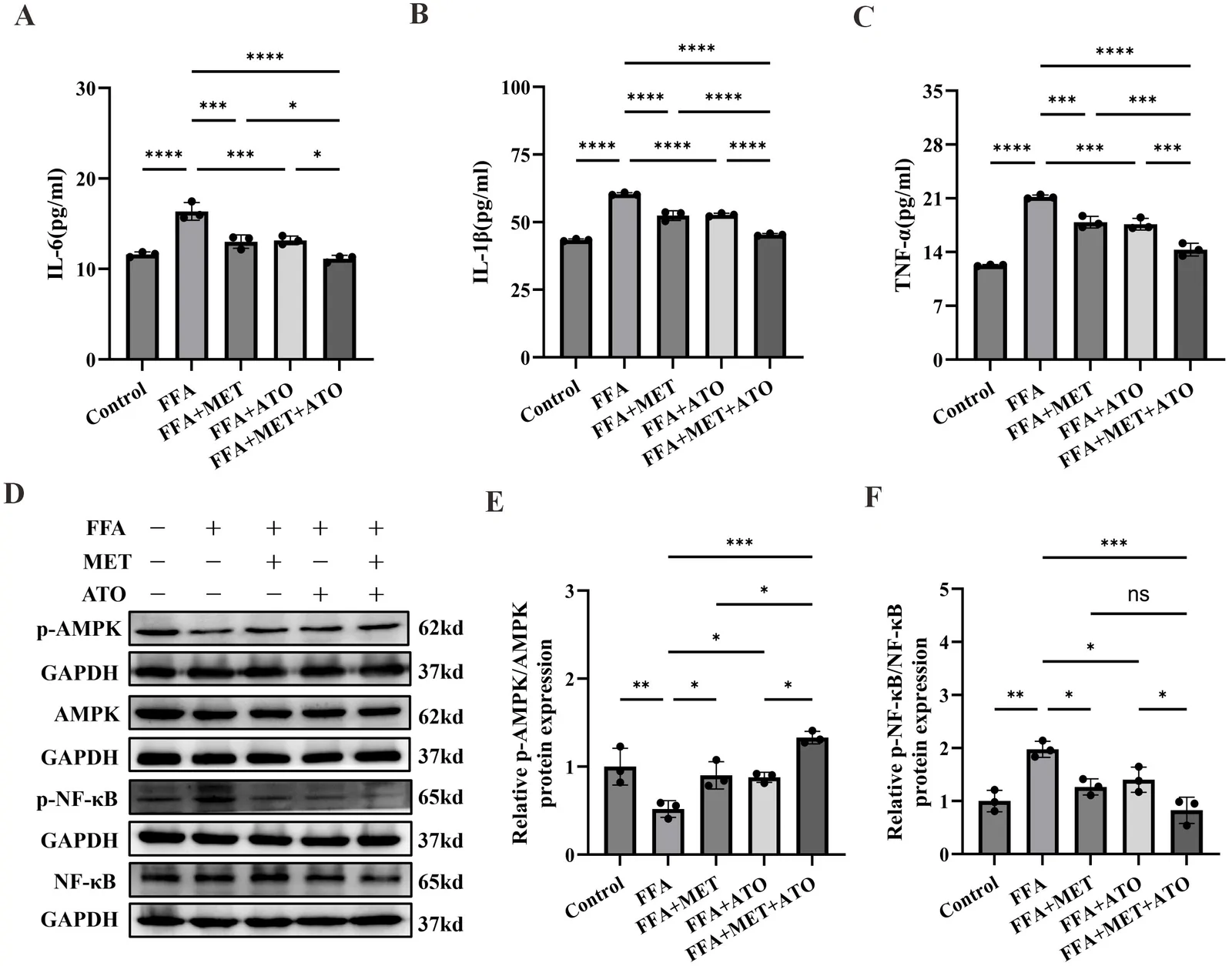
Depicts that combined metformin and atorvastatin treatment reduces FFA-induced inflammatory cytokine production in HepG2 cells. **(A–C)** ELISA quantification of supernatant IL-6, IL-1β, and TNF-α. **(D–F)** Relative phosphorylated expression of AMPK and NF‑κB quantified via Western blot analysis. Data are expressed as mean ± SEM (n = 3). *P < 0.05, **P < 0.01, ***P < 0.001, ****P < 0.0001.

Immunoblot analysis was carried out to examine the activation profile of the AMPK/NF-κB cascade, aiming to further uncover the intrinsic molecular regulatory mechanism. Relative to blank control cells, free fatty acid incubation markedly lowered the p-AMPK/AMPK protein ratio and simultaneously raised the p-NF-κB/NF-κB ratio. In contrast, combined MET and ATO treatment significantly restored AMPK phosphorylation and suppressed NF-κB phosphorylation (**Figures 2D–F**). The data imply that the anti-inflammatory response to the combined regimen involves upregulation of AMPK and downregulation of NF-κB signaling.

### 3.3 AMPK/NF-κB pathway activation mediates the alleviating effects of combined metformin and atorvastatin on FFA-induced lipid metabolic dysfunction and inflammation

HepG2 cells were preincubated with Compound C, an AMPK suppressor, prior to drug intervention to verify whether the protective action of co-administered MET and ATO relies on the AMPK/NF-κB signaling cascade. Results from Oil Red O histochemical staining and biochemical quantitative detection revealed that Compound C largely eliminated the combined treatment’s inhibition of intracellular lipid deposition, TG content, and TC content (**Figures 3A–D**). Consistently, Western blot detection indicated that the downregulation of SREBP-1 and HMGCR, as well as the elevated expression of CPT1A and PPARα induced by combined treatment was substantially abolished after Compound C incubation (**Figures 3E–I**).

**Figure 3:**
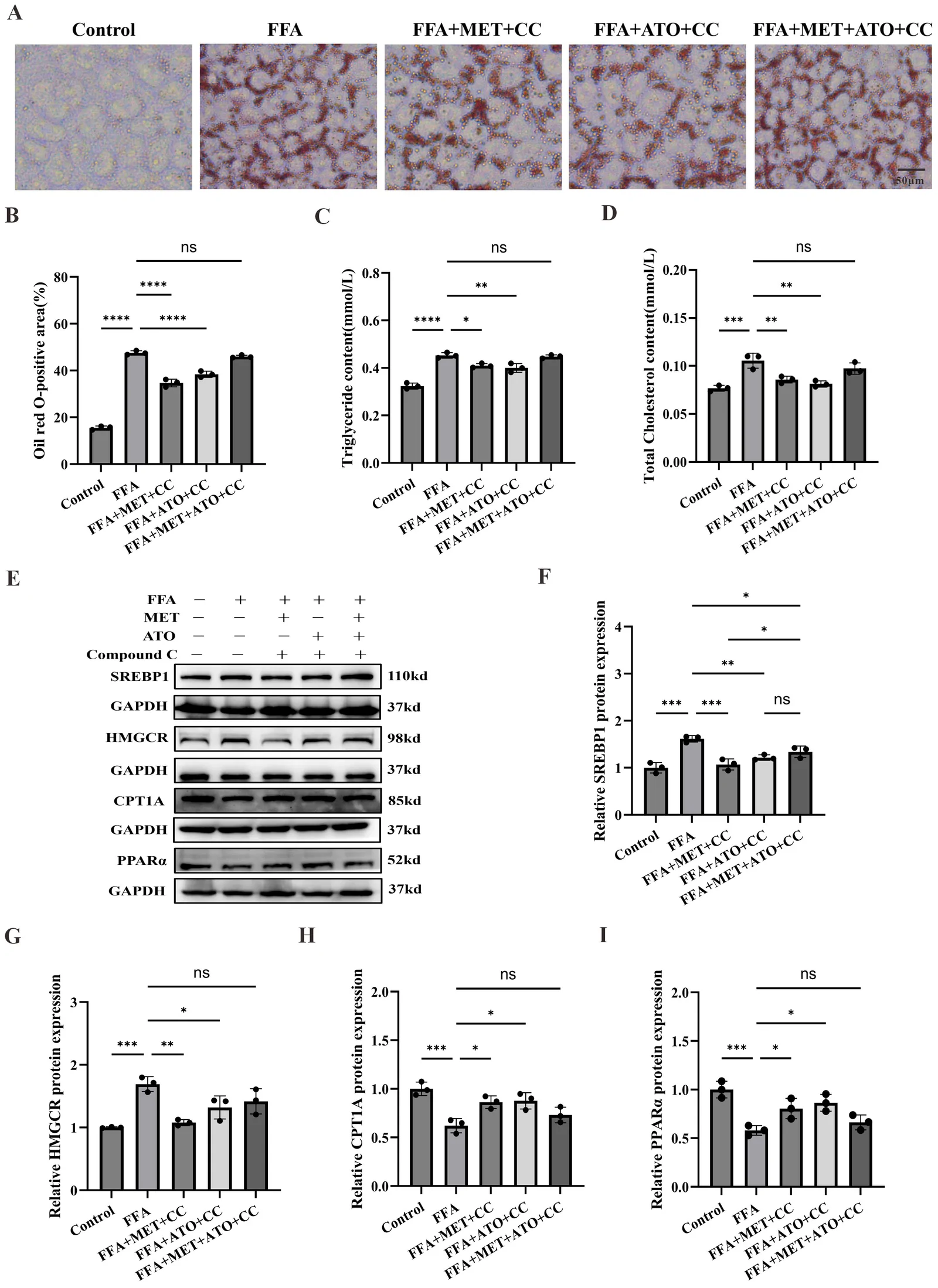
Depicts that combined metformin and atorvastatin treatment attenuates lipid synthesis in HepG2 cells through AMPK-dependent mechanisms. **(A)** Oil Red O staining micrograph following AMPK inhibitor treatment (40× objective; scale bar = 50 μm). **(B–D)** Statistical quantification for Oil Red O staining results as well as intracellular TG and TC contents. **(E–I)** Western blotting for SREBP1, HMGCR, CPT1A, and PPARα. All data are mean ± SEM (n = 3). *P < 0.05, **P < 0.01, ***P < 0.001, ****P < 0.000, ns, no statistical significance.

Furthermore, blocking AMPK activity greatly weakened the elevation of the p-AMPK/AMPK protein ratio and recovered the upregulated p-NF-κB/NF-κB level induced by FFA incubation (**Figures 4D–F**). Additional enzyme-linked immunosorbent assay data illustrated that the AMPK inhibitor Compound C substantially abrogated the ability of dual MET and ATO intervention to curb the production of pro-inflammatory cytokines IL-6, TNF-α and IL-1β (**Figures 4A–C**).

**Figure 4:**
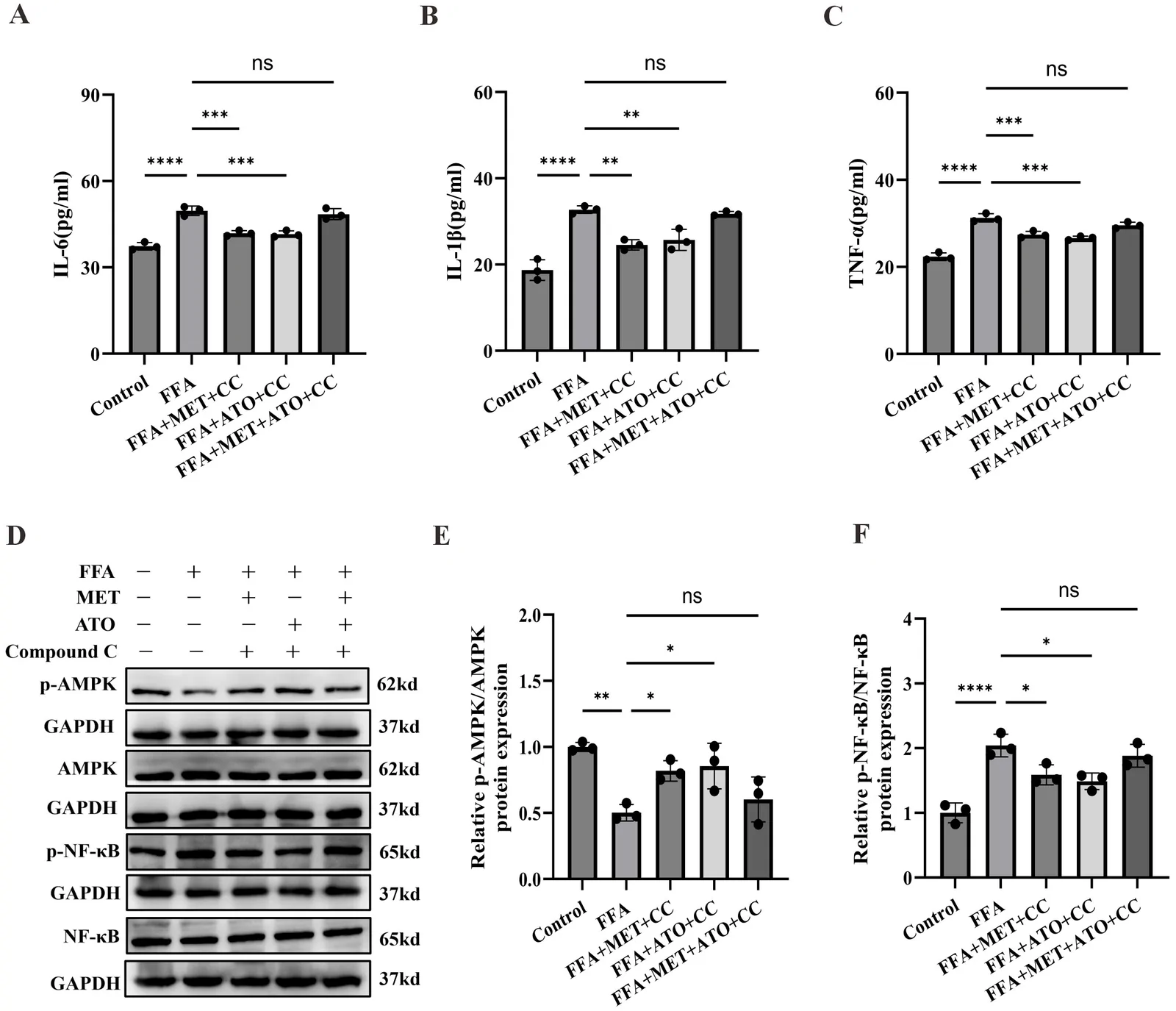
The joint administration of metformin and atorvastatin exerts inhibitory effects on inflammation in HepG2 cell lines through modulation of the AMPK/NF-κB signaling pathway. **(A–C)** Cytokine (IL-6, IL-1β, TNF-α) levels in culture supernatants via ELISA. **(D–F)** Immunoblot analysis showing the p-AMPK/AMPK and p-NF-κB/NF-κB protein ratios. All datasets are displayed as mean ± SEM (n = 3). *P < 0.05, **P < 0.01, ***P < 0.001, ****P < 0.0001, ns, no statistical significance.

Taken together, all experimental outcomes support that the dual-drug regimen of MET and ATO exerts protective regulation over lipid metabolism and inflammatory reactions primarily through triggering AMPK activation, which further suppresses NF-κB signal transduction.

### 3.4 Combined metformin and atorvastatin suppresses HFD/CCl_4_-induced body weight elevation and improves insulin resistance in mice

For characterizing the systemic metabolic regulatory efficacy of MET and ATO, assessments covering body weight, feed consumption, GLU, INS and HOMA-IR were conducted on murine models of MASH-like established via combined HFD and CCl_4_ intervention. Relative to the normal control cohort, the HFD-fed mouse group presented remarkable rises in indicators, including body weight, GLU, INS as well as HOMA-IR (**Figures 5A–C, E–G**). Monotherapy with MET or ATO elicited partial relief of these metabolic abnormalities, and combination therapy brought about much more prominent therapeutic benefits. No statistically significant differences in food consumption were found across all experimental groups (**Figure 5D**), suggesting that the observed reduction in body weight following combined treatment was not mediated by altered feeding behavior.

**Figure 5:**
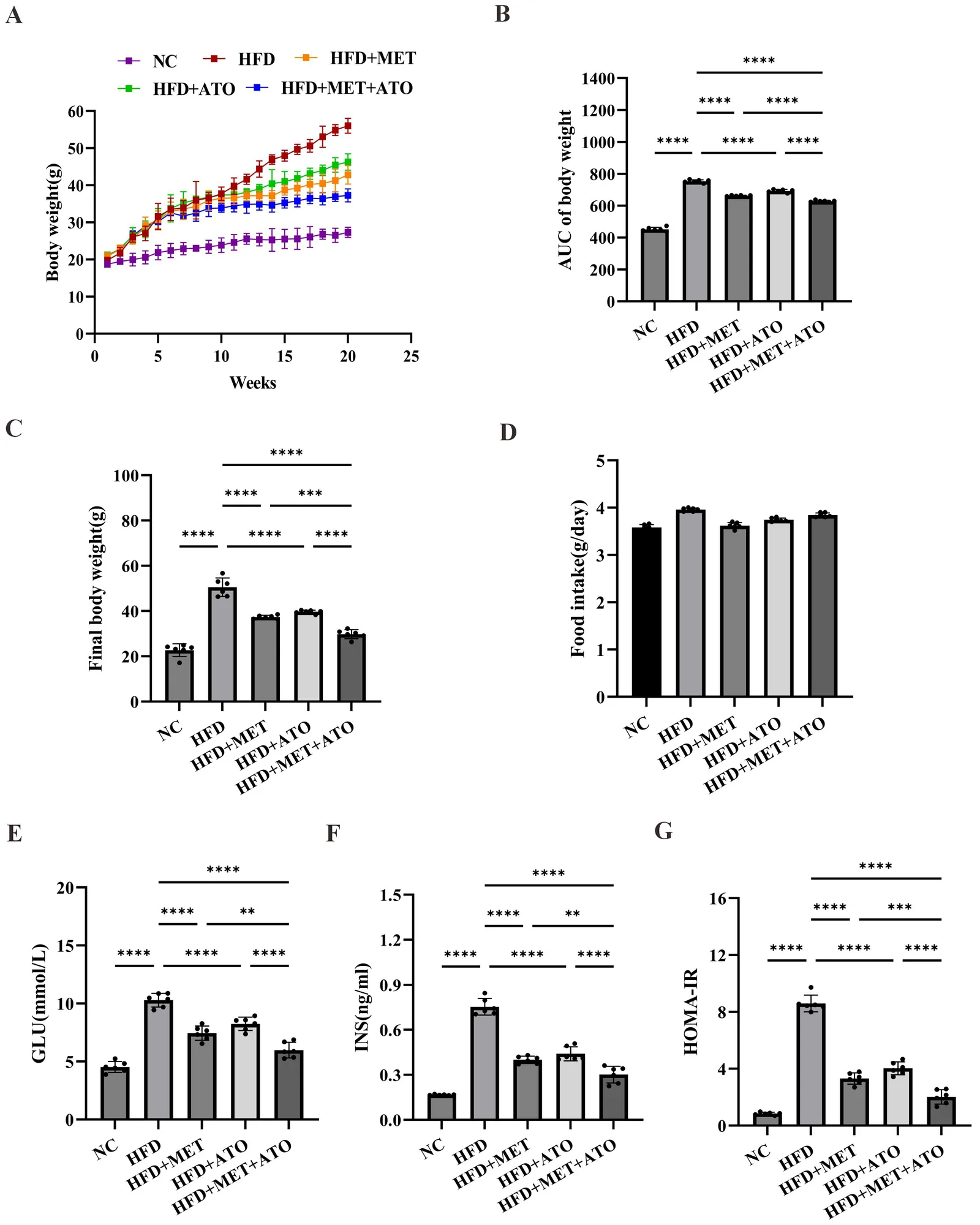
Combined treatment with metformin plus atorvastatin alleviates body weight increase and insulin intolerance triggered by a high-fat diet in mice. **(A)** Weekly body weight fluctuations during the 20-week intervention. **(B)** Area under the curve (AUC) calculated from body weight records of each experimental group. **(C)** Final body weight values. **(D)** Average daily food intake. **(E)** Fasting blood glucose (GLU). **(F)** Fasting serum insulin (INS). **(G)** HOMA-IR index. Experimental outcomes are shown as mean ± SEM (n = 6). **P < 0.01, ***P < 0.001, ****P < 0.0001.

Consistent with the above observations, dual intervention with MET and ATO generates potent improvements in metabolic disturbances linked to obesity as well as insulin resistance within HFD/CCl_4_-established MASH-like murine models.

### 3.5 Combined metformin and atorvastatin treatment attenuates hepatic steatosis and improves lipid metabolism in HFD/CCl_4_-induced MASH-like mice

To explore the regulatory impacts of MET combined with ATO on liver lipid metabolism, liver morphology, histological lipid accumulation, serum lipid profiles, hepatic lipid contents, and lipid metabolism-related proteins were assessed. In contrast to the control cohort, mice receiving HFD plus CCl_4_ intervention developed enlarged and pale liver tissues, which is a typical sign of serious hepatic fatty degeneration. In contrast, livers from mice receiving the combined treatment displayed a more normal gross appearance with a dark reddish color (**Figure 6A**). Oil Red O staining results further verified massive lipid droplet buildup within the HFD/CCl_4_ group, whereas lipid deposition was markedly reduced following MET or ATO monotherapy and was most effectively attenuated by the combined treatment (**Figure 6B**). Matching the earlier observations, mice subjected to HFD and CCl_4_ intervention displayed drastic elevations in liver index and the intrahepatic contents of TG and TC. These parameters were significantly reduced by both monotherapies, and the combination treatment brought about the most dramatic reduction (**Figures 6C–F**).

**Figure 6:**
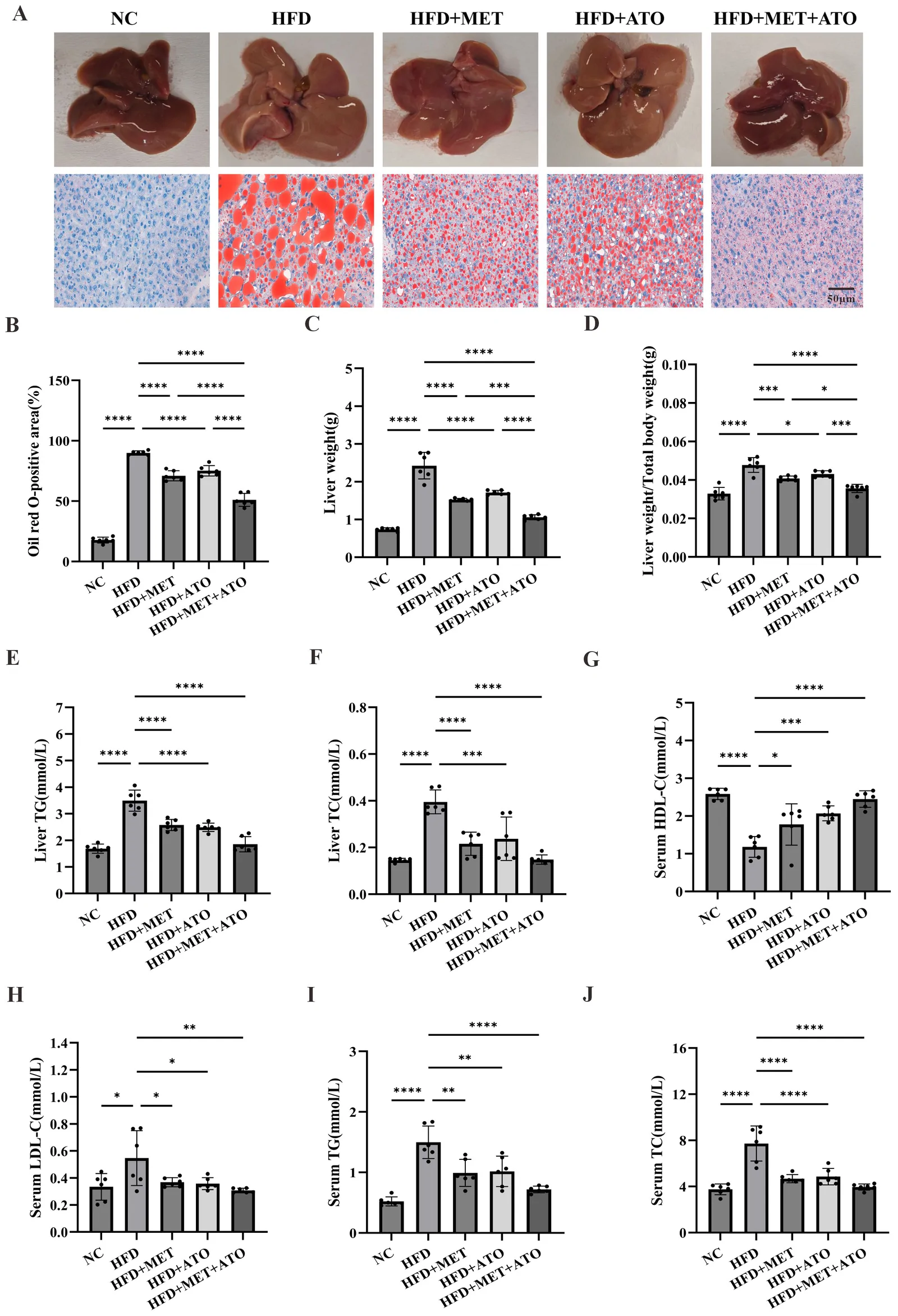
Combined MET and ATO treatment alleviates hepatic steatosis in MASH-like mice. **(A)** Typical macroscopic liver photographs alongside Oil Red O staining micrographs of hepatic sections in all groups (20× magnification, scale bar = 50 μm). **(B)** Quantitative detection of Oil Red O staining intensity. **(C, D)** Liver weight and liver-to-body weight ratio. **(E, F)** Hepatic TG and TC contents. **(G–J)** Serum levels of HDL-C, LDL-C, TG, and TC. Values are mean ± SEM (n = 6). *P < 0.05, **P < 0.01, ***P < 0.001, ****P < 0.0001.

Serum biochemical analysis showed that the HFD/CCl_4_ group exhibited significantly elevated LDL-C, TG, and TC levels, accompanied by decreased HDL-C level. Combined MET and ATO treatment produced greater improvements in these lipid parameters than either monotherapy (**Figures 6G–J**). Western blot testing further validated that the HFD combined with CCl_4_ intervention notably upregulated the lipogenic factors SREBP-1 and HMGCR, accompanied by suppressed expression of fatty acid oxidation critical proteins CPT1A and PPARα. These alterations were markedly reversed by the combined treatment (**Figures 7A–F**). Taken together, all murine experimental evidence supports that dual MET and ATO intervention exerts potent mitigating effects on hepatic fat accumulation, thereby recovering lipid metabolic balance in HFD/CCl_4_-triggered MASH-like animal models.

**Figure 7:**
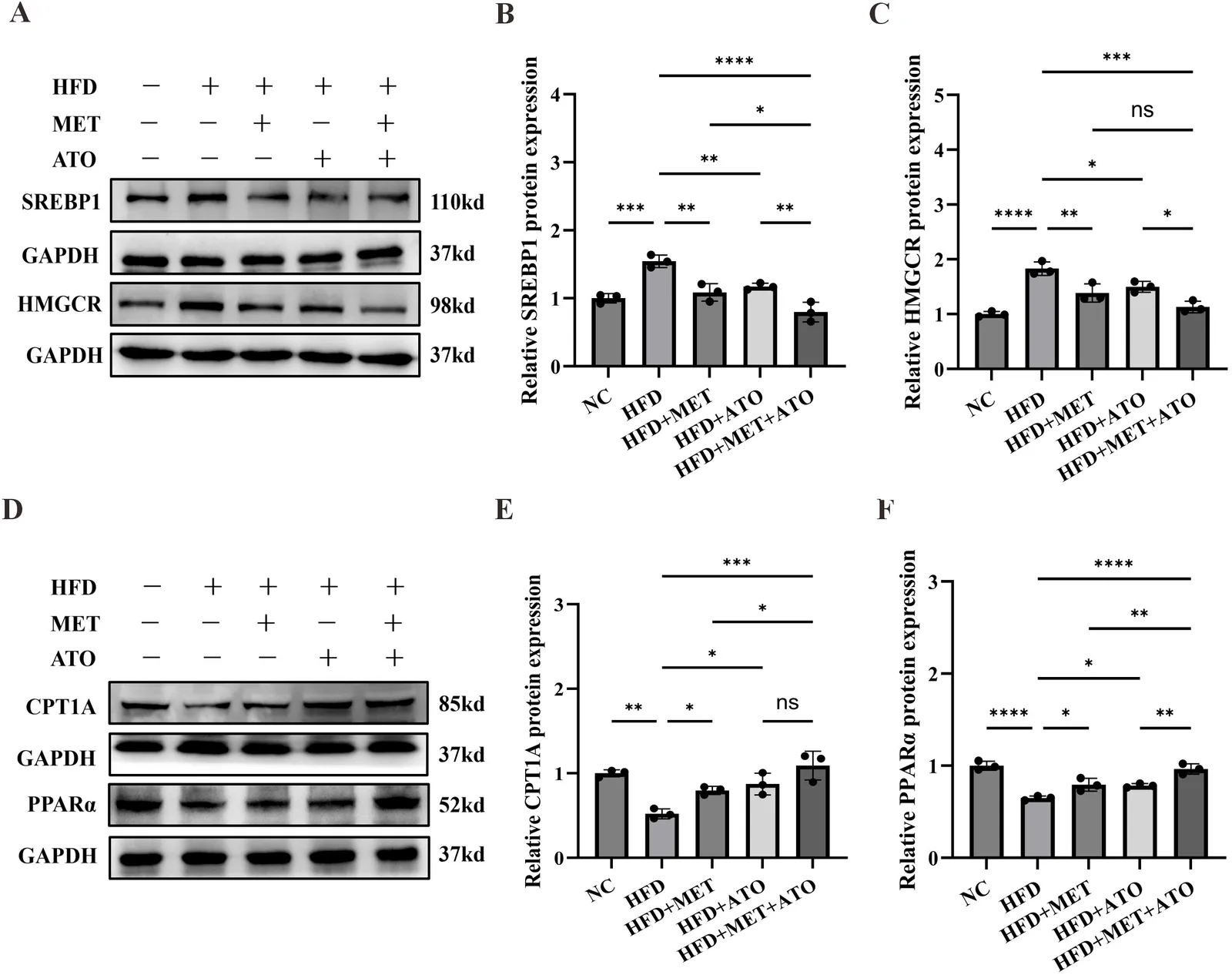
Influences of metformin-atorvastatin cotreatment on hepatic lipid metabolic protein expression in MASH-like murine models. **(A–C)** Western blot analysis of the lipogenic proteins SREBP1 and HMGCR in liver tissues. **(D–F)** Immunoblot detection of the fatty-acid-oxidation regulators CPT1A and PPARα. Data are mean ± SEM (n = 3). *P < 0.05, **P < 0.01, ***P < 0.001, ****P < 0.0001, ns: no statistical significance.

### 3.6 Combined metformin and atorvastatin treatment ameliorates liver injury and hepatic inflammation through modulation of the AMPK/NF-κB signaling pathway

For additional exploration into how dual MET and ATO therapy regulates liver damage and the inflammatory response, multiple detections were performed, including hepatic pathological morphology, liver function parameters, pro-inflammatory cytokine levels and the activation characteristics of AMPK/NF-κB signal transduction. According to H&E staining observation, murine liver tissues subjected to combined HFD and CCl_4_ stimulation displayed severe hepatic pathological abnormalities: vacuolar degeneration of parenchymal hepatocytes (yellow arrows), massive inflammatory cell infiltration (green arrows), and focal necrotic hepatocytes featuring karyorrhexis (silver arrows) (**Figure 8A**). These pathological alterations were substantially alleviated following MET or ATO treatment and were most effectively improved by the combined treatment. Consistently, the HFD/CCl_4_ group presented a prominent elevation in NAFLD Activity Score (NAS), whereas the combination therapy induced a striking drop in this index (**Figure 8B**). Biochemical quantification of serum specimens detected striking increases in ALT and AST contents within the HFD/CCl_4_ mouse cohort, a typical marker of hepatocellular impairment. Dual MET and ATO intervention exerted stronger inhibitory effects on the two enzymatic indicators relative to MET or ATO administered separately (**Figure 8C**). Likewise, enzyme-linked immunosorbent assay data illustrated that the HFD plus CCl_4_ model group displayed prominent accumulation of circulating IL-6, IL-1β and TNF-α. Dual-drug intervention yielded superior suppressive activity against the three inflammatory factors relative to individual MET or ATO administration (**Figures 8D–F**). Western blot analysis further demonstrated that HFD/CCl_4_ feeding markedly decreased the p-AMPK/AMPK ratio while increasing the p-NF-κB/NF-κB ratio. In contrast, combined MET and ATO treatment significantly restored AMPK phosphorylation and suppressed NF-κB phosphorylation (**Figures 8G–I**). Taken together, these findings suggest that combined MET and ATO treatment alleviates hepatic injury and inflammation to a considerable extent, and that this effect is associated with enhanced AMPK pathway activation and restrained downstream NF-κB signaling cascades.

**Figure 8:**
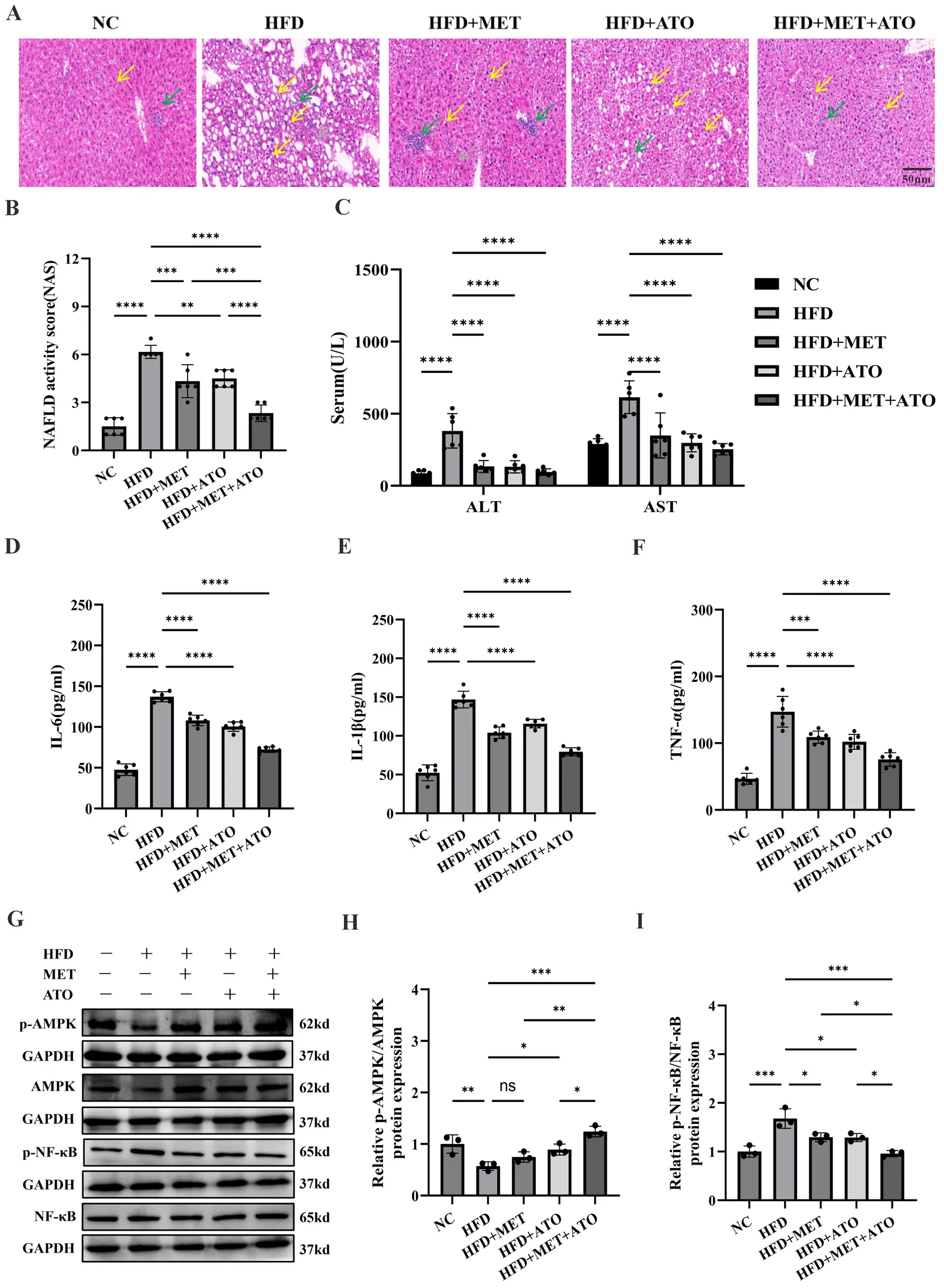
Combined MET and ATO therapy improves liver function and attenuates hepatic inflammation in MASH-like mice by activating AMPK and consequently inhibiting NF-κB signaling. **(A)** Typical H&E staining micrographs of liver tissue slices (20× magnification, scale bar = 50 μm). Yellow arrows mark lipid vacuoles, green arrows show lymphocyte infiltration, while gray arrows highlight hepatocellular necrosis. **(B)** NAFLD activity score (NAS) is calculated based on three indicators: hepatic steatosis, inflammatory lesions and hepatocellular ballooning degeneration. **(C)** Serum ALT and AST levels. **(D–F)** ELISA measurements of serum IL-6, IL-1β, and TNF-α. **(G–I)** Western blot profiles of p-AMPK/AMPK and p-NF-κB/NF-κB. Results of panels **(B–F)** are expressed as mean ± SEM (n = 6, animal-derived data), while those of panels **(H–I)** correspond to mean ± SEM across three independent Western blot tests. *P < 0.05, **P < 0.01, ***P < 0.001, ****P < 0.0001, ns, no statistical significance.

## 4 Discussion

Despite the established benefits of MET and ATO in metabolic disorders, their combined effects on MASH remain unexplored. Simultaneously improving lipid metabolism and inflammation is considered an effective strategy against MASLD. Our results showed that combined MET and ATO treatment significantly attenuated hepatic steatosis and inflammation in both mouse and cell models, accompanied by AMPK activation, NF-κB suppression, and reduced inflammatory cytokines. The AMPK-NF-κB axis appears to mediate the therapeutic promise of the combined MET and ATO regimen in MASH.

Disordered lipid metabolism is a fundamental feature of MASLD. Hepatic lipid balance is sustained by four processes: uptake, *de novo* lipogenesis, fatty acid oxidation, and lipid export (21). Disruption of this balance leads to triglyceride accumulation and hepatic steatosis (22). Our study showed that combined MET and ATO simultaneously suppressed lipogenesis and enhanced fatty acid oxidation. SREBP-1 controls *de novo* lipogenesis, while HMGCR is the rate-limiting enzyme in cholesterol synthesis and the target of statins (23, 24). MET activates AMPK to suppress SREBP-1 activity, whereas ATO inhibits HMGCR. Concurrently, increased CPT1A and PPARα expression indicated enhanced fatty acid oxidation. These complementary actions account for the reduced hepatic lipid deposition observed in our models, suggesting that the combination restores lipid homeostasis through complementary regulation of synthesis and oxidation. This aligns with reports that AMPK activation suppresses SREBP-1-mediated lipogenesis and that statins reduce hepatic steatosis (25, 26). Our study extends these observations by demonstrating that concurrent MET and ATO administration produces greater effects than either monotherapy, a feature not previously explored in MASH research.

Persistent mild inflammation constitutes another key factor fueling the advancement of MASLD. Pro-inflammatory cytokines (IL-6, TNF-α, and IL-1β) are released upon NF-κB axis activation, which is initiated by lipotoxicity-driven oxidative stress and mitochondrial impairment (27). The above pro-inflammatory substances will further worsen fat deposition in liver tissue and disrupt normal metabolic function, thereby establishing a self-perpetuating cycle linking lipotoxicity and inflammation (28). In this study, combined MET and ATO treatment significantly reduced circulating and cellular inflammatory cytokine levels while suppressing NF-κB phosphorylation. These findings suggest that improvement of lipid metabolism by the combined treatment may alleviate lipotoxicity-associated inflammatory activation, whereas simultaneous suppression of inflammatory signaling may further contribute to restoration of metabolic homeostasis. Accordingly, simultaneous modulation of lipid metabolic pathways and inflammatory reactions serves as the core mechanism responsible for the stronger liver-protective capacity delivered by co-administration of MET and ATO. Notably, a recent study also demonstrated that inhibiting lipid accumulation and inflammatory responses effectively ameliorates NASH, which is consistent with our observations of reduced lipid deposition and inflammation in the HFD/CCl_4_-induced MASH-like model (29).

AMPK is well acknowledged as a core modulator governing intracellular energy metabolism, and it also participates in the coordinated regulation of lipid turnover and inflammatory signal transduction (30). Stimulated AMPK can restrain endogenous fat synthesis alongside accelerating fatty acid β-oxidation; existing research also verifies it blocks NF-κB-mediated inflammatory cascades via diverse downstream molecular pathways (31). In the present study, combined MET and ATO treatment significantly increased AMPK phosphorylation while reducing NF-κB activation in both cellular and animal models. Furthermore, pharmacological inhibition of AMPK with Compound C largely abolished the favorable impacts of the combined treatment on lipid metabolism, inflammatory cytokine production, and NF-κB activation, supporting the involvement of the AMPK-NF-κB signal cascade in mediating these protective effects. This finding is consistent with prior studies showing that MET improves hepatic steatosis in an AMPK-dependent manner in rodent models, and that statins exert pleiotropic effects partly through AMPK activation (32, 33). While the present study still has several limitations, our study is the first to combine cellular and animal models to verify that MET‑ATO cotreatment effectively activates the AMPK‑NF‑κB pathway, inhibiting lipid deposition and inflammation. These results provide a new theoretical basis for combined therapies against MASH. Apart from local liver actions, accumulating data underscores the role of the spleen–liver axis in connecting systemic immune dysregulation to regional hepatic lipid metabolism in the context of MASLD (34). Serving as an immunological monitor, the spleen perceives metabolic stress and shuttles pro-inflammatory cells and cytokines to the liver directly by means of portal blood flow. Co-treatment with MET and ATO was observed to diminish the secretion of pro-inflammatory mediators including IL-6, TNF-α and IL-1β, and normalize lipid homeostasis marked by SREBP-1, CPT1A and PPARα. These beneficial outcomes probably stem from increased AMPK phosphorylation and the repression of NF-κB phosphorylation. This dual regulatory effect may break the vicious loop linking splenic immune priming and hepatic damage, thereby conferring systemic protection along the spleen–liver axis. These findings suggest that the spleen–liver axis may represent an important dimension through which combined treatment exerts its systemic benefits in MASLD. Because the spleen was not directly assessed in our study, this interpretation remains mechanistic and warrants further validation.

Additionally, the present investigation has several noteworthy limitations. First, our MASH-like model was established with 60% HFD plus CCl_4_, differing from standard diet-only MASLD protocols (e.g., ~45% HFD for 24 weeks). Human MASLD arises predominantly from dietary and metabolic disturbances. While this combined scheme rapidly induces advanced MASH-like characteristics (steatosis and inflammation), it includes CCl_4_-triggered hepatocyte injury and oxidative stress and may not fully reproduce the complete natural history of MASLD. Hence, additional studies with conventional diet-induced MASLD models that better mimic human disease progression are warranted to validate our data (35). Besides, systematic fibrosis analysis was absent from this study. Additional measurements of fibrotic parameters in future work will enable a more complete evaluation of the combined MET and ATO intervention against MASLD-associated fibrosis. Secondly, mechanistic validation of the AMPK signaling pathway was performed only in the cellular model using Compound C. Moreover, since the study did not include an FFA+CC-alone control group, we cannot fully eliminate the possibility that Compound C exerts independent influences on lipid and inflammatory parameters. Although our results strongly indicate that AMPK serves as a key molecular mediator underlying this combined therapy, additional experiments including an FFA+CC-alone control group are needed to confirm that Compound C specifically abolishes AMPK-linked protective actions. Thirdly, this work lacks *in vivo* evidence validating the AMPK/NF-κB pathway, and the animal studies were restricted by a modest sample size (n = 6 per group). Without adopting factorial design or widely accepted synergy assessment tools (e.g., CI analysis or the Bliss independence model) to examine MET–ATO interactions, we could only identify additive effects. For this reason, cautious interpretation of these mechanistic inferences is required. Further work featuring enlarged sample sizes to ensure sufficient statistical power, alongside genetic approaches (liver-specific AMPK knockout or overexpression models), is necessary to corroborate the AMPK/NF-κB axis and construct a customized statistical system for assessing *in vivo* synergy. Fourthly, while this research centers on the AMPK–NF-κB signaling cascade, multiple other pathways linked to lipid turnover, oxidative damage and autophagy regulation — such as SIRT1, Nrf2 as well as mTOR-associated signaling networks — are likely to partake in the combined therapeutic outcomes of MET combined with ATO, which deserves deeper exploration in follow-up experiments (36, 37).Finally, because this study was conducted using cellular and animal models, the therapeutic efficacy and safety of combined MET and ATO treatment require further validation in well-designed clinical studies.

## 5 Conclusions

In conclusion, this study demonstrates that combined MET and ATO effectively ameliorates hepatic lipid accumulation, metabolic dysfunction, and inflammation in experimental MASH, with superior efficacy over monotherapy. AMPK activation, followed by NF-κB blockade, appears to underlie these effects, leading to coordinated regulation of lipid metabolism and inflammation. Our findings provide experimental evidence supporting this combination strategy and identify the AMPK-NF-κB axis as a promising therapeutic target for MASH.

## Data Availability

The original contributions presented in the study are included in the article/**Supplementary Material**. Further inquiries can be directed to the corresponding author.
