# Supplementary material for: Metformin and atorvastatin ameliorate MASH via the AMPK/NF-κB pathway
Source: Front Endocrinol (Lausanne). 2026 Oct 12;17:1972846. doi: 10.3389/fendo.2026.1972846 (PMC13696279; doi:10.3389/fendo.2026.1972846)

Due to time constraints and limited antibody availability, detection of certain target proteins was performed by cutting the same PVDF membrane into multiple strips, incubating with corresponding antibodies, and developing separately. The uncropped original membrane images are presented below, with each membrane labeled with the target protein name, molecular weight markers, and the cropped areas used for quantification indicated by red boxes. All Western blot experiments were repeated independently at least three times, and representative membranes are shown.

The supplementary figures are organized as follows:

Supplementary Cellular experiments 1 – Uncropped membranes corresponding to Figure 1 in the main text (cellular steatosis-related proteins: SREBP-1, HMGCR, CPT1A, PPARα).

Supplementary Cellular experiments 2 – Uncropped membranes corresponding to Figure 2 in the main text (cellular inflammation and pathway-related proteins: p-AMPK, AMPK, p-NF-κB, NF-κB).

Supplementary Inhibitor experiments 1 – Uncropped membranes corresponding to Figure 3 in the main text (lipid metabolism-related proteins after inhibitor treatment: SREBP-1, HMGCR, CPT1A, PPARα).

Supplementary Inhibitor experiments 2 – Uncropped membranes corresponding to Figure 4 in the main text (pathway-related proteins after inhibitor treatment: p-AMPK, AMPK, p-NF-κB, NF-κB).

Supplementary Animal experiments 1 – Uncropped membranes corresponding to Figure 7 in the main text (hepatic lipid metabolism-related proteins in animals: SREBP-1, HMGCR, CPT1A, PPARα).

Supplementary Animal experiments 2 – Uncropped membranes corresponding to Figure 8 in the main text (hepatic pathway and inflammation-related proteins in animals: p-AMPK, AMPK, p-NF-κB, NF-κB).

Cellular experiments1

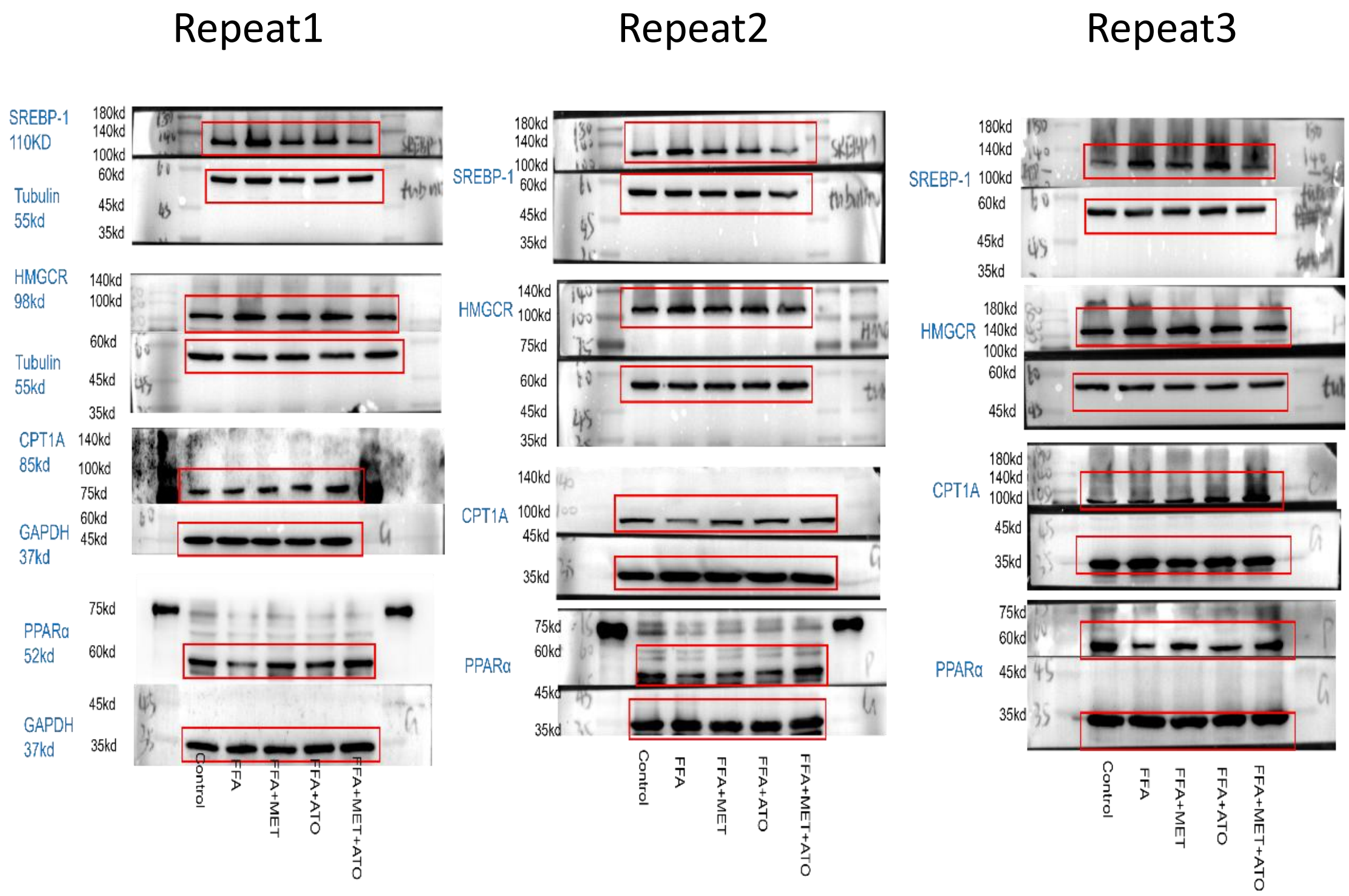

Cellular experiments2

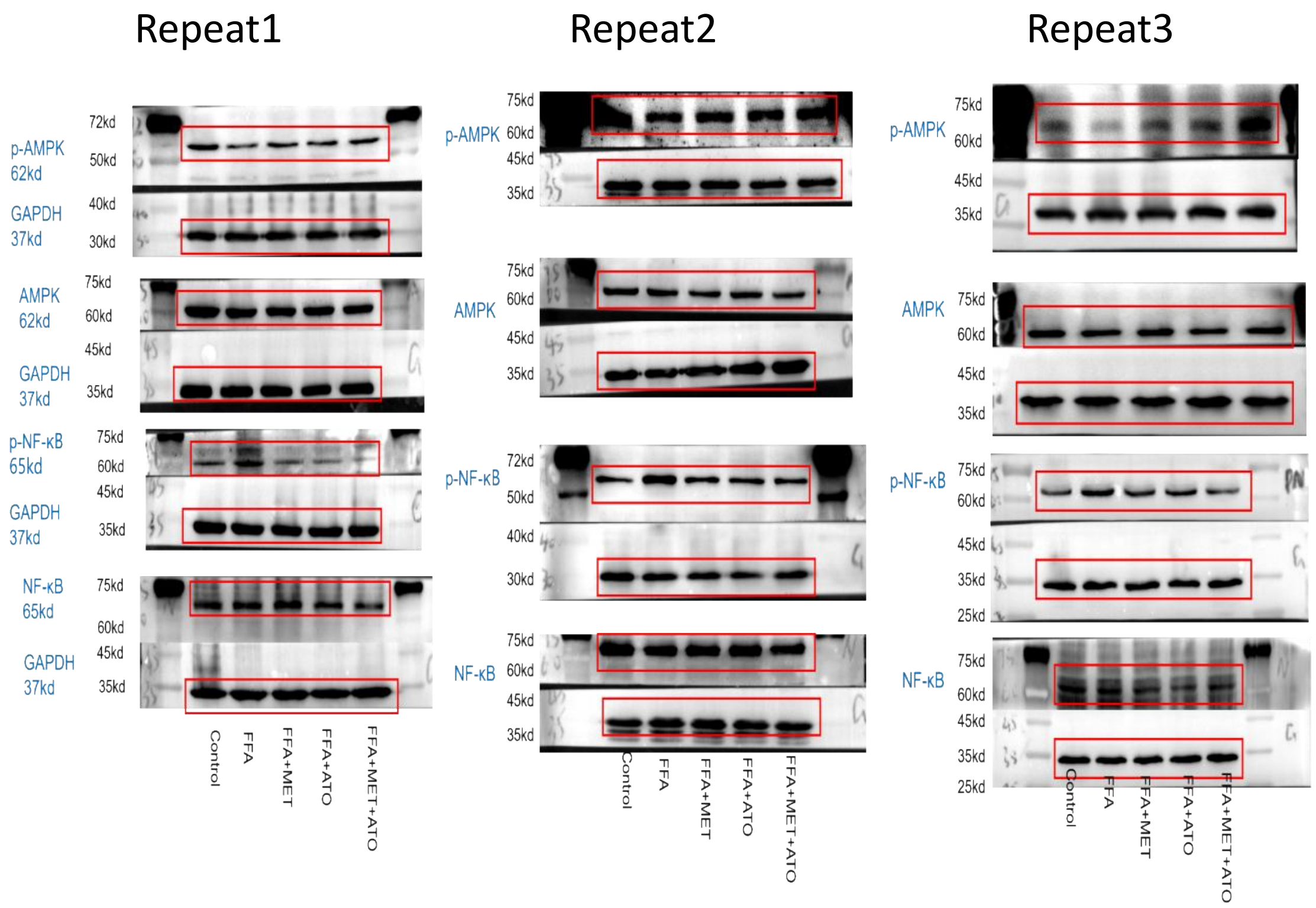

Inhibitor experiments1

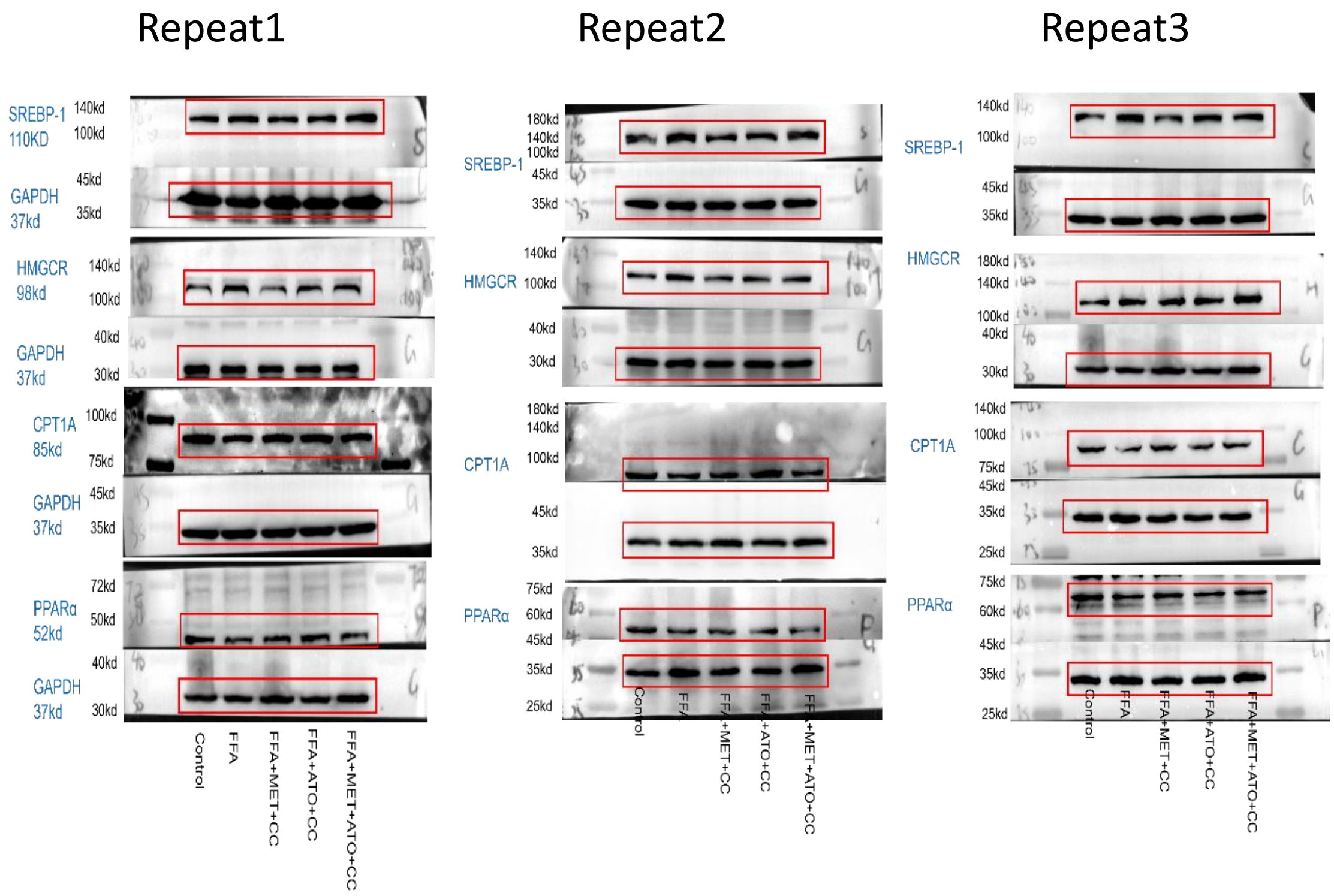

Inhibitor experiments2

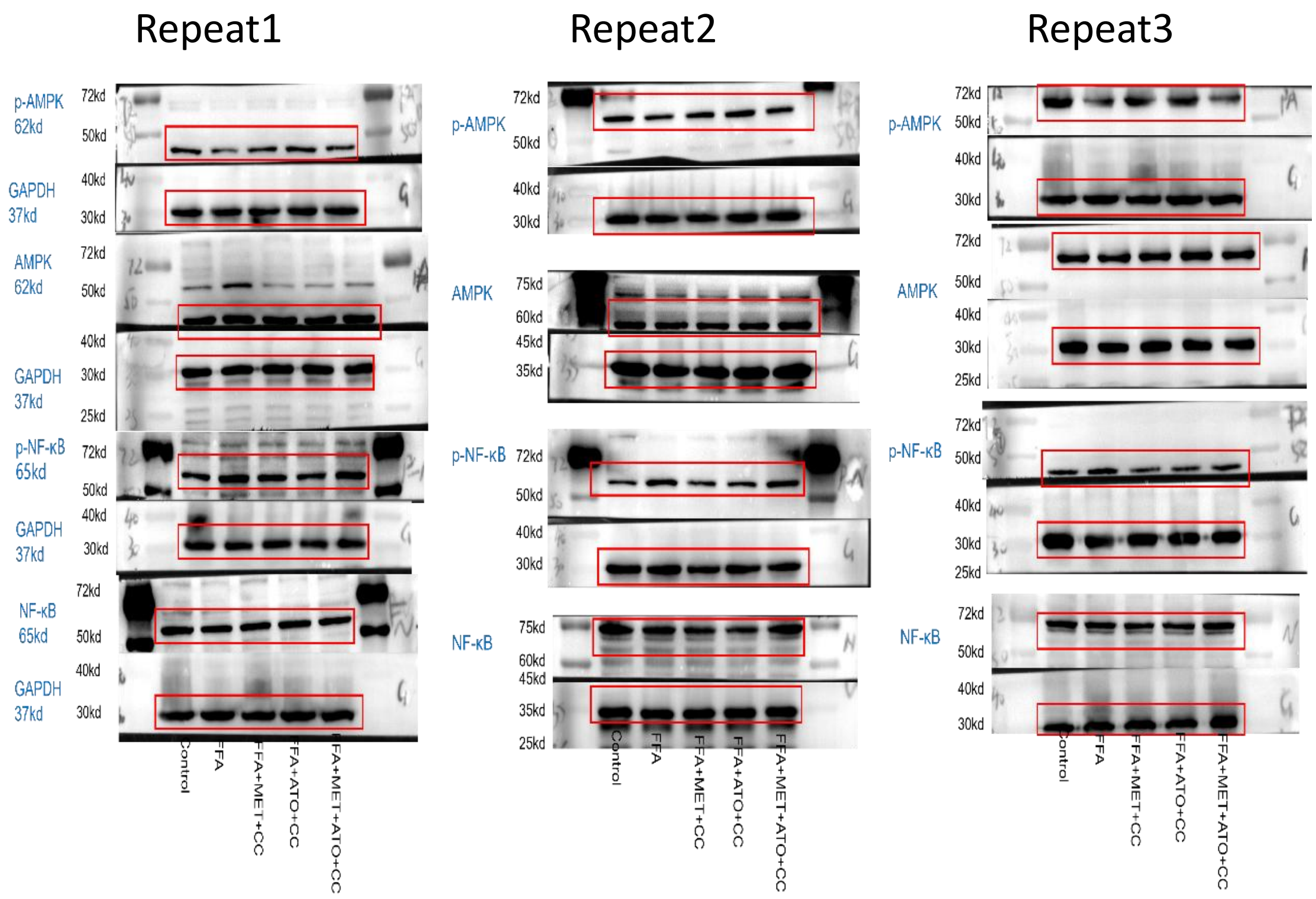

Animal experiments1

Repeat1 Repeat2 Repeat3

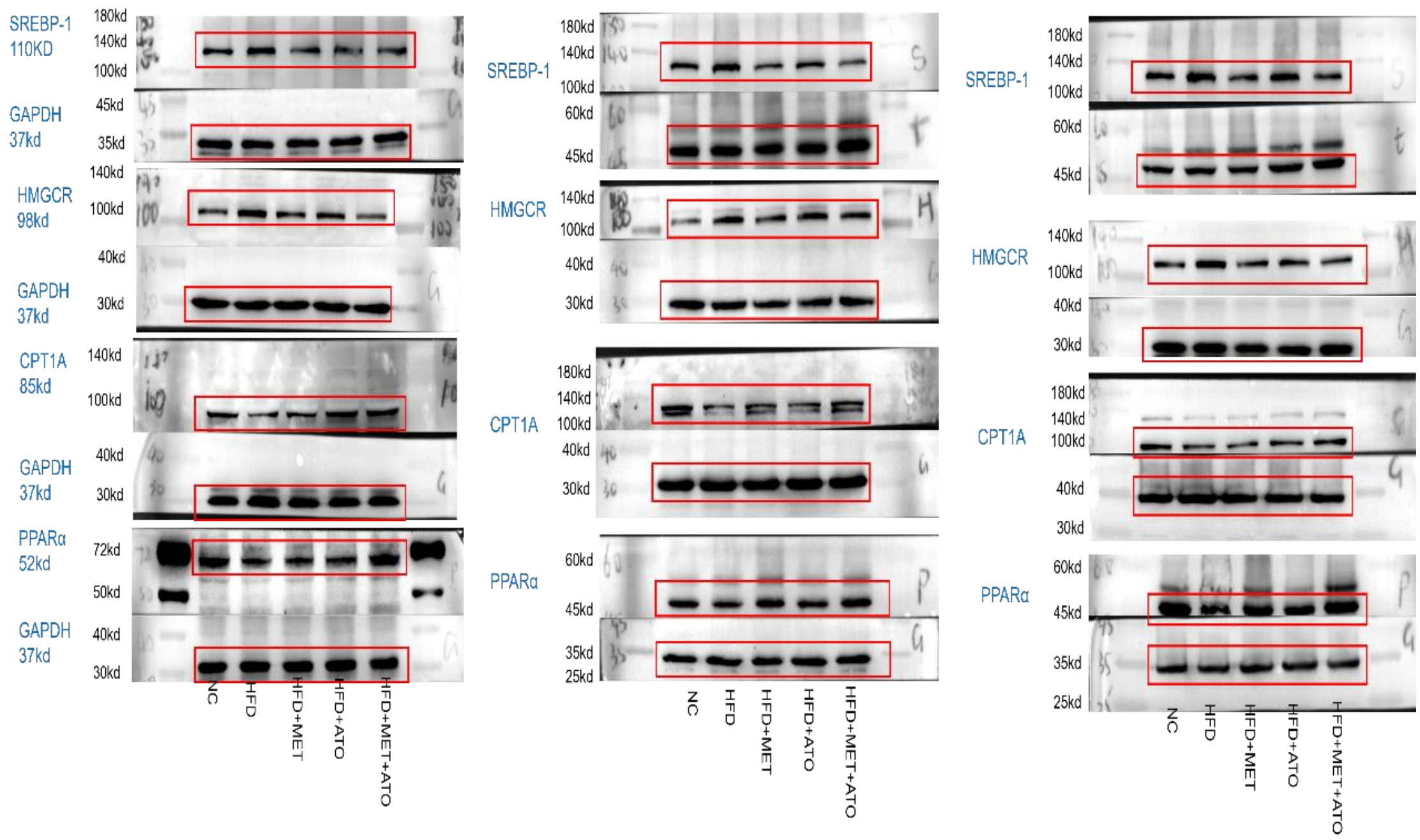

Animal experiments2

Repeat1 Repeat2 Repeat3

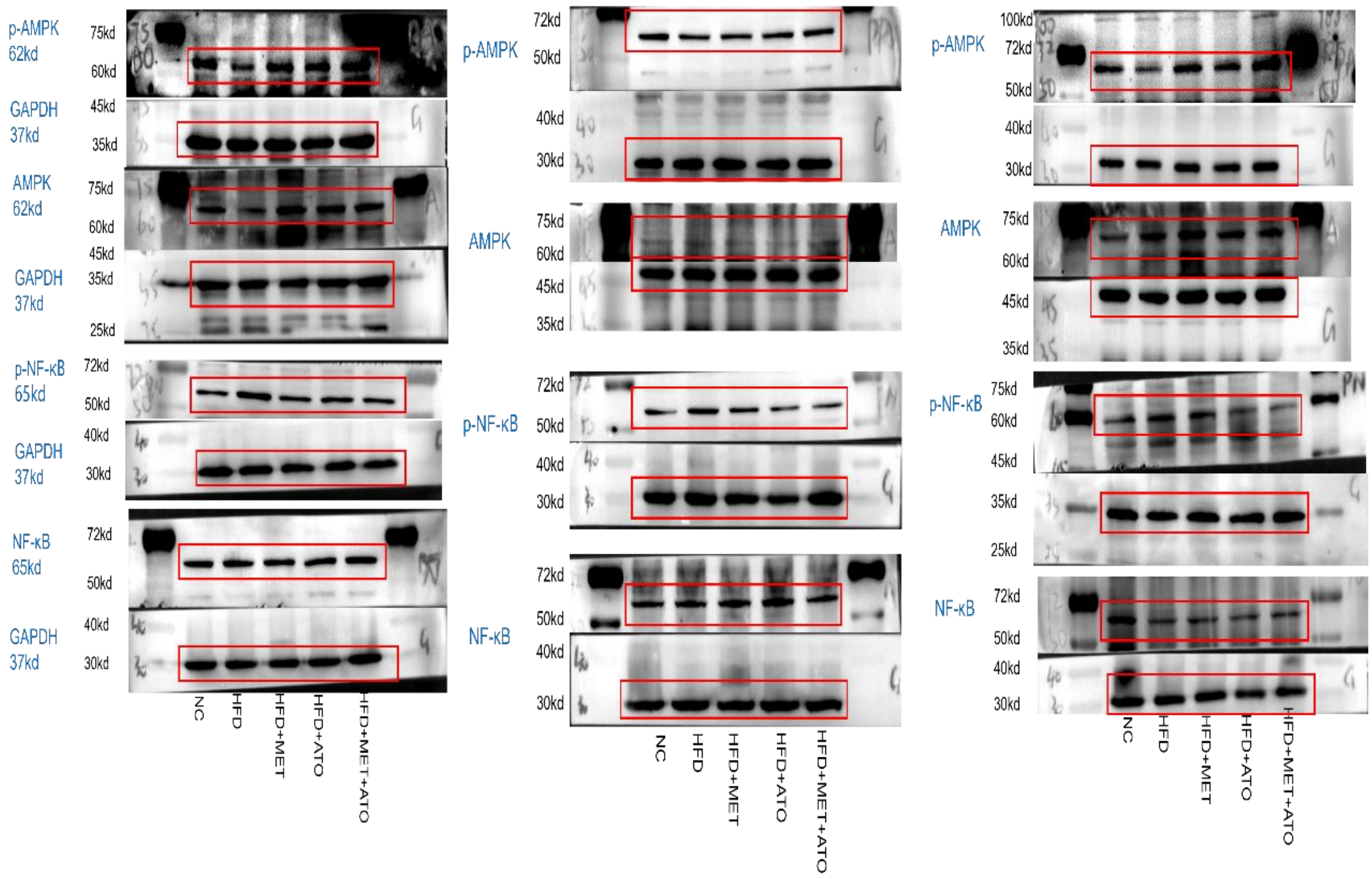

Supplement: Supplementary file 1 [file DataSheet1.pdf]
