# Supplementary material for: Metformin and atorvastatin ameliorate MASH via the AMPK/NF-κB pathway
Source: Front Endocrinol (Lausanne). 2026 Oct 12;17:1972846. doi: 10.3389/fendo.2026.1972846 (PMC13696279; doi:10.3389/fendo.2026.1972846)
Supplement: Supplementary file 2 [file DataSheet2.pdf]

Supplementary Material

Supplementary Figure 2

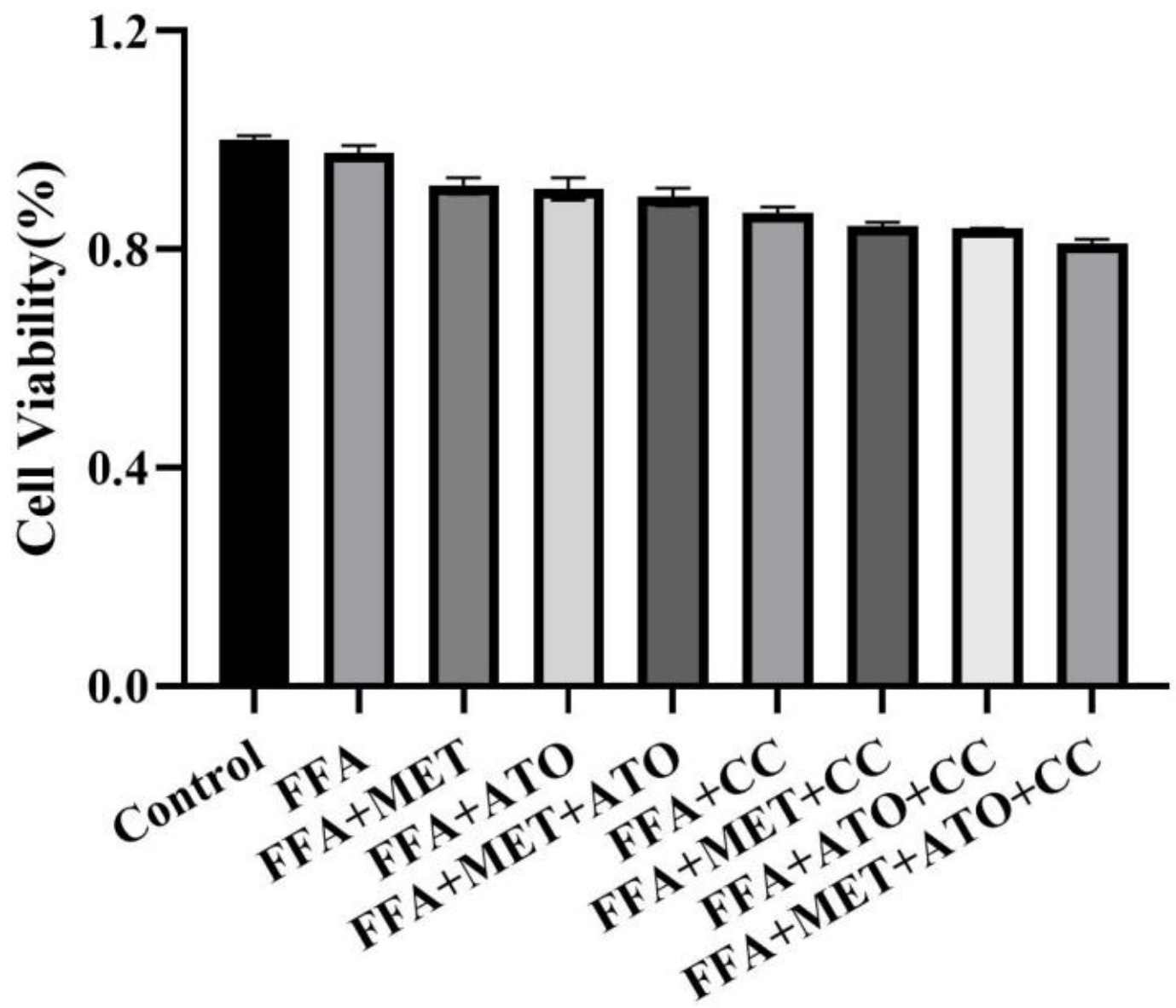

Supplementary Figure 2. Cell viability in all experimental groups. Cell viability was assessed by CCK-8 assay in Control, FFA, FFA+MET, FFA+ATO, FFA+MET+ATO, and Compound C-containing groups. Cell viability remained above 80% in all groups. Data are presented as mean ± SD (n = 3).
